# Editorial: Genotype–phenotype correlations in neurodegenerative diseases: From clinical features to neuroimaging signatures

**DOI:** 10.3389/fneur.2022.1100953

**Published:** 2022-12-16

**Authors:** Pilar M. Ferraro, Nicola Spotorno, Eoin Finegan, Frederic Sampedro, Francesca Caso

**Affiliations:** ^1^Neurology Unit, IRCCS Ospedale Policlinico San Martino, Genoa, Italy; ^2^Clinical Memory Research Unit, Department of Clinical Sciences, Malmö, Lund University, Lund, Sweden; ^3^Computational Neuroimaging Group, Biomedical Sciences Institute, Trinity College Dublin, Dublin, Ireland; ^4^Movement Disorders Unit, Neurology Department, Hospital de la Santa Creu i Sant Pau, Barcelona, Spain; ^5^Biomedical Research Institute (IIB-Sant Pau), Barcelona, Spain; ^6^Centro de Investigación en Red-Enfermedades Neurodegenerativas (CIBERNED), Barcelona, Spain; ^7^Neurology Unit, IRCCS San Raffaele Scientific Institute, Milan, Italy

**Keywords:** genetic, neurodegeneration, neuroimaging, motor neuron diseases, multiple sclerosis, progressive supranuclear palsy, fragile X-associated tremor/ataxia syndrome, Rett syndrome

Genetic factors play a key role in the pathogenesis of several neurodegenerative disorders, both as monogenic causes of inheritable disease forms and as modifiers of susceptibility to sporadic phenotypes.

Over the last two decades tremendous advances have been made in identifying the genetic underpinnings of multiple neurodegenerative conditions, and such developments are critically informing both new disease models and more targeted therapeutic strategies.

With the growth of gene-focused clinical trials it is imperative to better characterize genotype–phenotype correlations and improve our knowledge of the underlying pathological mechanisms.

In this context, neuroimaging provides a particularly promising tool to investigate the neurobiological processes mediating genotype–phenotype associations. Different imaging modalities are providing complementary information on many mechanisms involved in the pathophysiological cascades, including regional vulnerability to atrophy, microstructural damage and alterations in functional activity.

Of note, recent studies also suggest that these changes are detectable even in the asymptomatic/presymptomatic disease phases, opening unprecedented possibilities for unraveling compensatory mechanisms and developing preventive therapeutic strategies.

The goal of this Research Topic was to give an overview of clinical and neuroimaging studies focusing on the complex interplay between genetic signatures and phenotypic expressions in neurodegenerative diseases.

Starting from the description of clinical features characterizing genetic disease forms, the usefulness of different imaging modalities to unravel the underlying pathological mechanisms was further evaluated and commented.

The Research Topic included five articles and provided an overview of neuroimaging approaches for investigating genotype-phenotype correlations.

Chai et al. applied a combination of consensus weighted gene co-expression network (WGCNA) and single-sample gene set enrichment (ssGSEA) analyses to compare the gene co-expression networks of gray matter (GM) and white matter (WM) lesions in multiple sclerosis (MS), observing both significant similarities and differences.

In particular, the authors identified common gene expression patterns in signaling pathways related to the immature and activated B-cell and the central memory CD4^+^ *T-*cell, while differentially expressed genes were mainly distributed in the CD56 bright natural killer cell pathway. These findings provided an example of the relevance of transcriptomics approaches for better clarifying the pathological mechanisms sustaining MS onset and progression.

In another contribution, Wang et al. explored the prevalence and clinical relevance of novel neuroradiological markers in fragile X-associated tremor/ataxia syndrome (FXTAS) premutation carriers using quantitative susceptibility mapping (QSM).

The authors evaluated the presence of globus pallidus abnormal T2-signals in addition to the classic middle cerebellar peduncle (MCP) sign, showing that such characteristic features are associated with greater cognitive impairment and faster age-related iron depletion and variability, providing preliminary evidence that the interaction between MCP and pallidal T2-signal changes may underlie the heterogeneous clinical manifestations of FXTAS.

Ruiz-Barrio et al. contributed with a review on clinical and neuroimaging features of genetic progressive supranuclear palsy (PSP) syndromes and further proposed a diagnostic algorithm that may help the genetic diagnosis when there is clinical suspicion of a monogenic disease form.

The collected data and the suggested diagnostic workflow have the potential for a significant impact both on clinical practice and therapeutics development, since the recognition of larger case series with well-defined mutations, combined with a specific evaluation of their clinical and neuroimaging features may add new insights into the pathophysiological mechanisms leading to neuronal damage in these complex conditions.

The work by Spinelli et al. provided an example of such potential in motor neuron diseases (MNDs). By combining voxel-based morphometry (VBM) and diffusion tensor imaging (DTI) in a relatively large sample of *TARDBP* mutation carriers, the authors were able to identify a distinctive parietal pattern of cortical atrophy and greater damage of motor and extra-motor WM tracts, providing preliminary evidence that TDP-43 pathology due to *TARDBP* mutations may cause deeper GM and WM morphologic alterations in this pathological spectrum.

Finally, the review by Kong et al. further highlighted the relevance of neuroimaging investigations in genetic neurodevelopmental disorders, particularly in Rett syndrome (RTT) with MECP2 mutation.

Specifically, the authors provided a detailed overview of the diverse pathological signatures of the syndrome as revealed by the application of distinct imaging modalities, and further discussed the associations of different metrics with clinical manifestations, confirming the potential of neuroimaging application for elucidating the complex interplay between genes, abnormalities in neurotransmitter pathways, and clinical symptoms.

In summary, over a wide spectrum of diverse neurological conditions, the articles included in this Research Topic have provided converging evidence on the relevance of investigating genetic disease forms to unravel the biological complexity and clinical heterogeneity of neurodegenerative and neurodevelopmental disorders.

The common focus on the application of diverse neuroimaging modalities has highlighted the utility of multimodal approaches to gain complementary information on the diverse pathological mechanisms involved in the onset and progression of such complex disorders, opening new possibilities for improved disease modeling strategies.

In conclusion, through the collection of original research articles and reviews focused on clinical and neuroimaging signatures of genotype-phenotype correlations, we believe this Research Topic has contributed to advancing our understanding of the complex interaction between genetic underpinnings, phenotypic manifestations and underlying pathological mechanisms across multiple neurological disorders.

Future studies will advance this research field, possibly through the evaluation of larger case series and an increasing focus on presymptomatic disease phases, finally enabling the translation of the obtained findings into the development of early personalized therapeutic strategies.

## Author contributions

All authors listed have made a substantial, direct, and intellectual contribution to the work and approved it for publication.

